# The big CGRP flood - sources, sinks and signalling sites in the trigeminovascular system

**DOI:** 10.1186/s10194-018-0848-0

**Published:** 2018-03-12

**Authors:** Karl Messlinger

**Affiliations:** Institute of Physiology and Pathophysiology, Friedrich-Alexander-Universität Erlangen-Nürnberg, Universitätsstr. 17, 91054 Erlangen, Germany

## Abstract

**Background:**

Calcitonin gene-related peptide (CGRP) has long been a focus of migraine research, since it turned out that inhibition of CGRP or CGRP receptors by antagonists or monoclonal IgG antibodies was therapeutic in frequent and chronic migraine. This contribution deals with the questions, from which sites CGRP is released, where it is drained and where it acts to cause its headache proliferating effects in the trigeminovascular system.

**Results:**

The available literature suggests that the bulk of CGRP is released from trigeminal afferents both in meningeal tissues and at the first synapse in the spinal trigeminal nucleus. CGRP may be drained off into three different compartments, the venous blood plasma, the cerebrospinal fluid and possibly the glymphatic system. CGRP receptors in peripheral tissues are located on arterial vessel walls, mononuclear immune cells and possibly Schwann cells; within the trigeminal ganglion they are located on neurons and glial cells; in the spinal trigeminal nucleus they can be found on central terminals of trigeminal afferents. All these structures are potential signalling sites for CGRP, where CGRP mediates arterial vasodilatation but not direct activation of trigeminal afferents. In the spinal trigeminal nucleus a facilitating effect on synaptic transmission seems likely. In the trigeminal ganglion CGRP is thought to initiate long-term changes including cross-signalling between neurons and glial cells based on gene expression. In this way, CGRP may upregulate the production of receptor proteins and pro-nociceptive molecules.

**Conclusions:**

CGRP and other big molecules cannot easily pass the blood-brain barrier. These molecules may act in the trigeminal ganglion to influence the production of pronociceptive substances and receptors, which are transported along the central terminals into the spinal trigeminal nucleus. In this way peripherally acting therapeutics can have a central antinociceptive effect.

## Preface

The title of the EHF Lecture was inspired by the famous allegorical woodcut “The Great Wave of Kanagawa” by Katsushika Hokusai (1760-1849) with the idea of two recent scientific and clinical phenomena that made calcitonin gene-related peptide (CGRP) popular in migraine research: first, the wave of CGRP that may flush our head during a migraine attack, and second, the hype about new options in migraine therapy by blocking the CGRP system. What is so special about this neuropeptide, a member of a family of peptides, which in a similar form is already functional in the most primitive chordate animals like Amphioxus [47], and which is now regarded as a key mediator in migraine headache; the plasma level of which has been used as a biomarker for migraine; and, when its actions are blocked, migraine attacks may be prevented or stopped?

### Discovery of CGRP and first functional findings

CGRP was firstly described as a splice product of the calcitonin gene in two well-recognized papers by Amara et al. [2] and Rosenfeld et al. [41]. They found calcitonin mRNA predominating in the rat thyroid, while mRNA for another peptide, which they named calcitonin gene-related peptide, predominated in the hypothalamus and other neural tissues. Shortly after these findings, Mason et al. [35] reported about the release of immunoreactive CGRP from cultured rat trigeminal ganglion cells, and Wiesenfeld-Hallin et al. [53] subsequently showed that dorsal root ganglion neurons and their putative terminals in the spinal dorsal horn were marked by CGRP immunofluorescence, partly co-stained by substance P immunofluorescence. A similar finding was also described for the trigeminal ganglion by Lee et al. [30]. Around the same time Edvinsson et al. [12] were the first to describe the potent relaxing effect of CGRP on feline cerebral arteries, an effect concomitant with an accumulation of cyclic adenosine monophosphate (cAMP). Consistent with this, Uddman et al. [52] demonstrated by immunohistochemistry and radioimmunoassay that CGRP containing nerve fibers surround these blood vessels. Shortly after these reports the same group showed clearly the important role of CGRP in cerebrovascular regulation, specifically as a function of trigeminal nerve fibers antagonizing local and sympathetic vasoconstrictory effects [36].

The following considerations about the release, draining and signalling of CGRP are restricted to the trigeminovascular system. This system constitutes a functional unit of intracranial blood vessels and their trigeminal innervation and is the most likely source of nociceptive events that lead to headaches [40].

### Sources for CGRP in the trigeminovascular system

**Fig. 1:**
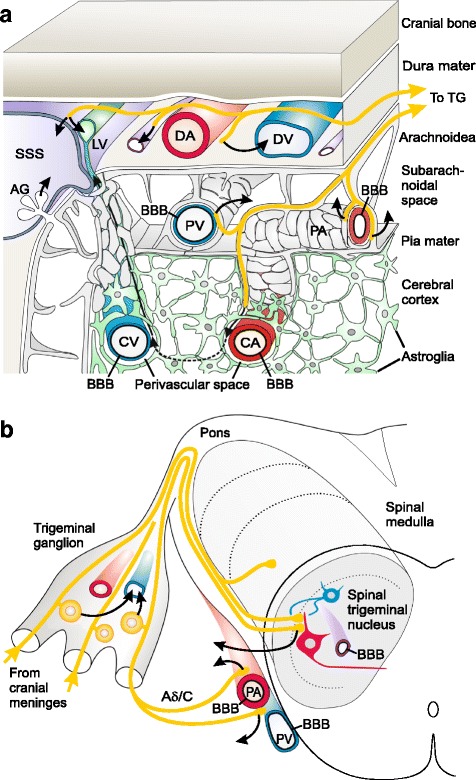
Scheme of sources and sinks for CGRP (curved arrows) in the trigeminovascular system, **a**meninges, **b** trigeminal ganglion and spinal trigeminal nucleus. CGRP released from trigeminal afferent fibers innervating dural arteries (DA) is most likely taken up from capillaries, venous vessels (DV) and possibly the superior sagittal sinus (SSS), and is transported with the blood stream into the internal jugular vein. The same may occur in the trigeminal ganglion, which is located outside the blood-brain barrier (BBB). However, a BBB is functional in pial arteries (PA) and veins (PV). Thus, CGRP released from perivascular afferent fibres cannot enter the vessels but diffuses into the surrounding cerebrospinal rooms, the subarachnoidal space or the cerebello-medullary cisterna (cisterna magna), respectively, where it can be found in the cerebrospinal fluid (CSF). Probably part of it is secreted through the arachnoid granulations (AG) into the SSS and appears secondarily also in the jugular blood. CGRP released from the central terminals of trigeminal afferents within the spinal trigeminal may move out of the medulla into the cisterna magna. Innervated cortical arterioles (CA) arising from penetrating pial arteries may also be innervated by trigeminal afferents. Here, CGRP may be released into the perivascular space between the vessel wall and the surrounding astroglial end-feet. Then it may be transported with the glymphatic flow through the brain tissue and collected in the venous perivascular spaces. CGRP may move together with the extracellular fluid within these spaces along the venous vessels through the subarachnoidal space to the dura mater, where it may be finally collected in lymphatic vessels (LV) accompanying the SSS

In the trigeminovascular system, the immunohistochemical identification of CGRP has been used to identify the sources, from which this neuropeptide is potentially released (Fig. 1). The cranial dura mater is innervated by CGRP immunoreactive nerve fibres running along the meningeal arteries and terminating at their branches but also between blood vessels in the connective tissue [38]. Stimulation of meningeal afferents by substances like capsaicin in an ex vivo preparation of the hemisected rodent head caused release of immunoreactive CGRP quantified by an ELISA [10]. This stimulation may render the smooth shape of CGRP-ir nerve fibres perl-like supposing a re-distribution of CGRP prior to its release [23]. Likewise, pial and intracerebral arteries are innervated by CGRP immunoreactive trigeminal afferent fibres [11]. An additional site of potential CGRP release in the trigeminal system is the trigeminal ganglion, which, in rodents and humans, contains CGRP immunoreactive neurons of mainly small diameter that make up nearly half of the neuronal population [17, 31]. Looking to the central nervous system, a dense plexus of CGRP immunoreactive fibres in the superficial layers of the spinal trigeminal nucleus represents the central terminals of these neurons [15]. From both isolated rodent trigeminal ganglia and medullary slices containing these terminals, considerable amounts of immunoreactive CGRP could be released by noxious agents like capsaicin [29]. The anti-migraine drug naratriptan did not significantly inhibit capsaicin-induced CGRP release from peripheral terminals but inhibited the release from brainstem slices suggesting a potential central effect of triptans, if they penetrate the blood brain barrier.

### Sinks for CGRP in the trigeminovascular system

During migraine attacks increased concentrations of CGRP have been found in the plasma of the internal jugular vein [13] but the intracranial sinks of the drained CGRP are not clearly known. It is assumed that CGRP is released from intracranial sources into the venous blood, most likely from activated primary afferents. An increase in jugular plasma CGRP was measured following topical application of depolarizing concentrations of potassium chloride (KCl) onto the exposed rat parietal dura mater [8] suggesting that CGRP released from dural afferent fibres is taken up into the meningeal venous system and transported via the sagittal sinus to the jugular vein (Fig. 1a). Interestingly, after infusion of a CGRP-binding L-RNA oligonucleotide, the Spiegelmer NOX-C89, CGRP has accumulated in the plasma, which may indicate that in an unbound state CGRP is rapidly eliminated from the blood plasma. Action potentials elicited through excitation of meningeal afferents certainly reach the somata within the trigeminal ganglion and may also cause CGRP release there, though we have only evidence from in vitro studies for this assumption [9]. However, since blood vessels supplying the ganglion do not have a blood-brain barrier (BBB), like those supplying the cranial dura [18], the released CGRP is also drained via the venous plasma and can contribute to the plasma CGRP values measured in jugular blood (Fig. 1b).

In contrast, pial blood vessels on the surface of the cerebral cortex and medulla oblongata are equipped with a BBB. It is likely that CGRP does not penetrate the BBB with the consequence that, when it is released from nerve fibres innervating these vessels, it cannot reach the vessel lumen but must diffuse into the surrounding perivascular space bordered by a layer of leptomeninx and finally into the cerebrospinal fluid (CSF) within the subarachnoid space (Fig. 1a, b). Indeed, five times higher concentrations of CGRP can be measured in the rat CSF compared to the plasma [8]. The CGRP concentration in the CSF could further be increased when noxious inflammatory mediators were injected into the ventricle system [24]. However, the administration of inflammatory mediators also increased levels of CGRP in jugular plasma, probably indicating that CGRP is rapidly absorbed together with the CSF through the arachnoid granulations extending into the superior sagittal sinus.

It is very likely, however, that the CSF is also the sink for CGRP from a different source, namely CGRP released from the activated central terminals of primary afferents in the spinal trigeminal nucleus (Fig. 1b). The medullary blood vessels are also equipped with a BBB that prevents inward diffusion of peptides. These peptides may diffuse to the surface of the medulla and into the surrounding CSF, as has been suggested by earlier radiographic measurements of neuropeptide release using a microprobe technique [43]. In line with this assumption, CGRP levels in the CSF were also increased by activating dural afferents with depolarizing KCl [8]. This increase was abolished by preceding anaesthesia of the trigeminal ganglion, suggesting that the increased CGRP appearing in the CSF was released by the central terminals of activated meningeal afferents.

In this context the potential importance of the recently described glymphatic system should be discussed as an additional sink for CGRP. This lymphatic-like system consists of a small extracellular space between the wall of intracerebral blood vessels and the astroglial end-feet surrounding the vessels. This space may be in continuation with the Virchow-Robin space of the penetrating arteries, although such a connection has not been confirmed by anatomical data [55]. However, tracer experiments in rodents suggest that CSF is driven from the subarachnoid space along the pial arteries into the perivascular space of intracerebral arterial vessels [26] from which fluid can move into and out of the astrocytes by aquaporines, ion channels and transporters [1]. Via the interconnected astrocytes a convective flow is assumed to move through the brain from the perivascular space of cerebral arteries to the perivascular space of venous vessels, and in this way substances accumulating in the brain can be washed into the venous glymphatic system [51]. The venous perivascular space may continue along the pial veins travelling through the subarachnoid space to the dura mater [25], where this drainage system is continued by lymphatic vessels along the sinus, and these are ultimately connected to the cervical lymphatic system [3]. Thus, if trigeminal peptidergic afferents innervating the penetrating cerebral arteries are activated, CGRP may be released into the perivascular space and transported via the glymphatic flow into the lymphatic system as a third compartment of CGRP accumulation (Fig. 1a). This issue is all the more interesting in light of recent evidence showing that perivascular spaces are collapsing during experimental cortical spreading depression, which is believed to be the pathophysiological correlate of the migraine aura [44].

### Signalling sites for CGRP in the trigeminovascular system

CGRP receptors are heteromers, composed of a seven transmembrane spanning protein called calcitonin receptor-like receptor (CLR), a single membrane-spanning protein called receptor activity-modifying protein 1 (RAMP1), and an intracellular component, the receptor component protein [28]. CGRP binds in a molecular pocket formed by CLR and RAMP1, thus immunohistochemical colocalization of both components can indicate the expression sites of functional CGRP receptors.

In the rodent cranial dura mater, immunofluorescence for both CLR and RAMP1 was found associated with arterial blood vessels, mononuclear cells (mast cells and probably macrophages) and nerve fibres [31]. High-power confocal images combining CLR and RAMP1 immunoreactivity and markers for axons and glia suggest that Schwann cells but not axons express CGRP receptors, although these findings have been questioned by another group, who found that CGRP receptor immunofluorescence is associated with myelinated A-fibre axons [19].

In the trigeminal ganglion of all species examined so far, apart from blood vessels, neurons of mainly middle sizes and glial cells (Schwann cells and satellite cells) have been found immunopositive for both CGRP receptor components [18, 31]. Likewise, the central processes of these neurons terminating in the outer layers of the spinal trigeminal nucleus and the accompanying glia also express these receptor proteins [15, 31]. While there is conflicting data regarding whether or not neuronal cell bodies in the spinal trigeminal nucleus express CGRP receptor proteins, there is agreement that trigeminal neurons projecting to the trigeminal brainstem are either expressing CGRP or CGRP receptor components [16, 31].

### Signalling of CGRP in the trigeminovascular system

**Fig. 2:**
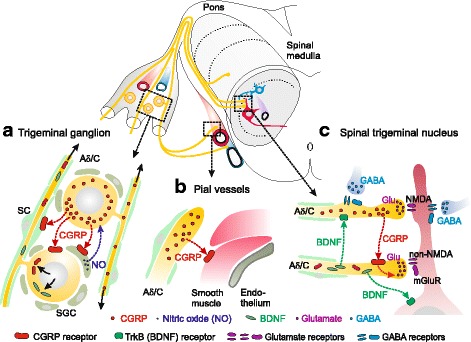
Scheme of signalling sites for CGRP in the trigeminovascular system. In the trigeminal ganglion (**a**) CGRP released from neurons may signal to neighbouring neurons, glial satellite cells (GSC) and possibly Schwann cells (SC) expressing CGRP receptors and can thus, via gene expression, influence the production of substances, e.g. nitric oxide (NO), brain-derived neurotrophic factor (BDNF) and CGRP receptor components. These (NO) may signal back to the neurons, or (BDNF, CGRP receptors) may be transported through the central extensions of trigeminal afferents (Aδ/C) to the spinal trigeminal nucleus. CGRP signalling at arterial vessels, e.g. the pial vessels of the spinal medulla (**b**), causes vasodilatation and increased blood flow. Within the spinal trigeminal nucleus (**c**) CGRP is released from central terminals of trigeminal afferents and signals most likely to other central terminals equipped with CGRP receptors, which may lead to increased neurotransmitter (glutamate) release and facilitation of nociceptive transmission. Release of BDNF may pre- and postsynaptically potentiate synaptic transmission. Neurons with inhibitory neurotransmitters (GABA) may counteract the pronociceptive synaptic processes

CGRP is regarded as the most potent vasodilator of intracranial arteries [14]. It has been long known that CGRP binding to its receptor on meningeal (dural and pial) vascular smooth muscle cells induces relaxation through increased intracellular cyclic adenosine monophosphate (cAMP) levels and thereby causing arterial vasodilatation [27] (Fig. 2b). This effect can be observed directly by video imaging, e.g. pial arteries on the medulla [54], and examined indirectly by recording meningeal or medullary blood flow [8]. A weak mast cell degranulating effect of CGRP indicated by histamine release was found in rat dura mater [45] but does probably not translate to human meninges [39]. The question of whether CGRP directly activates meningeal afferents or not has been largely refuted; CGRP does not cause firing nor rapid sensitization of meningeal afferents when it is locally administered onto the rodent dura mater, although application of cAMP analogs can indeed sensitize meningeal afferents [32, 33]. The failure of CGRP to activate meningeal afferents is probably due to the lack of functional CGRP receptors on the nerve fibres innervating the dura mater [31]. Even direct injection of CGRP or CGRP receptor antagonists into the rat trigeminal ganglion did not change the activity or mechanical sensitivity of neurons in the spinal trigeminal nucleus with meningeal afferent input [6]. Since systemic administration and iontophoretic application of CGRP receptor antagonists into the spinal trigeminal nucleus are well suited to decrease spinal trigeminal activity [49], we have concluded that CGRP is primarily activating trigeminovascular neurons at their synapses within the trigeminal nucleus [21, 37] (Fig. 2c).

However, these results were all based on short observation periods and did not regard long-term changes caused by activation of cAMP-dependent intracellular signalling mechanisms, which result in the upregulation of transcription factors like extracellular signal-regulated kinase (ERK) and cAMP response element binding protein (CREB) via phosphorylation of protein kinases [42]. Through these mechanisms a variety of receptor proteins like functional purinergic receptors (P2X3) have been shown to be upregulated in trigeminal cell cultures by CGRP application [20, 48]. These mechanisms may include signalling between neurons and glia (satellite cells), both of which may express CGRP receptors (Fig. 2a). For example, CGRP released from neurons could cause expression of inducible nitric oxide synthase (iNOS) in satellite cells to produce nitric oxide (NO) [34]. NO could in turn activate the MAP kinase pathway in neighbouring neurons resulting in a higher expression of CGRP, as it was shown in cell cultures [4]. Similar cross-signalling mechanisms involving NO may also occur in vivo leading to an upregulation of CGRP and CGRP receptor proteins, as evidenced by immunohistochemical findings [7, 46]. Especially interesting are proteins that are transported through the central processes of the ganglion cells to the spinal trigeminal nucleus, where they can be integrated into the presynaptic membrane (Fig. 2a, c). An example of this is brain-derived neurotrophic factor (BDNF), the expression of which is induced by CGRP in cultured trigeminal neurons [5]. BDNF released from central presynaptic terminals may act on pre- and postsynaptic tyrosine kinase (TrkB) receptors and strengthen nociceptive transmission [22]. Similarly, we assume that CGRP receptors integrated into the presynaptic membrane of central trigeminal terminals are activated by CGRP released from central terminals of other trigeminal afferents and this facilitates neurotransmitter (glutamate) release and thereby nociceptive transmission. Evidence for CGRP-evoked facilitation of central glutamate release comes from experiments on spinal cord slices [50]. The principles outlined above provide an explanation for the mechanism by which inhibition of the CGRP signalling system outside the BBB, i.e., particularly in the trigeminal ganglion, can dampen trigeminal nociception, although the main CGRP dependent pro-nociceptive effects may take place within the spinal trigeminal nucleus.

## Abbreviations

5-HT: 5-hydroxytryptamine (serotonin)
BBB: Blood-brain barrier
BDNF: Brain-derived neurotrophic factor
cAMP: Cyclic adenosine monophosphate
CGRP: Calcitonin gene-related peptide
CLR: Calcitonin receptor-like receptor
CREB: cAMP response element binding protein
CSF: Cerebrospinal fluid
ELISA: Enzyme-linked immuno sorbent assay
ERK: Extracellular signal-regulated kinase
iNOS: Inducible NO synthase
KCl: Potassium chloride
L-RNA: Ribonucleic acid made with (unnatural) L-ribose
MAP-kinase: Mitogen-activated protein kinase
mRNA: Messenger ribonucleic acid
NO: Nitric oxide
P2X3: Purinergic receptors
RAMP1: Receptor activity-modifying protein 1
TrkB: Tyrosine kinase receptor B, receptor for BDNF
